# Proximity Labeling-Based Identification of MGAT3 Substrates and Revelation of the Tumor-Suppressive Role of Bisecting GlcNAc in Breast Cancer via GLA Degradation

**DOI:** 10.3390/cells14020103

**Published:** 2025-01-12

**Authors:** Bowen Wang, Xin He, Yue Zhou, Zengqi Tan, Xiang Li, Feng Guan, Lei Lei

**Affiliations:** 1 Key Laboratory of Resource Biology and Biotechnology Western China, Ministry of Education, Provincial Key Laboratory of Biotechnology, College of Life Sciences, Northwest University, Xi’an 710069, China; 202010253@stumail.nwu.edu.cn (B.W.); yue.zhou@nwu.edu.cn (Y.Z.); 2 Department of Functional Laboratory, College of Laboratory Medicine, Dalian Medical University, Dalian 116044, China; hex01@dmu.edu.cn; 3 Institute of Hematology, School of Medicine, Northwest University, Xi’an 710069, China; zengqtan@nwu.edu.cn (Z.T.); xiangli@nwu.edu.cn (X.L.)

**Keywords:** bisecting GlcNAc, MGAT3 substrates, proximity labeling, GLA

## Abstract

Glycosylation plays a critical role in various biological processes, yet identifying specific glycosyltransferase substrates remains a challenge due to the complexity of glycosylation. Here, we employ proximity labeling with biotin ligases BASU and TurboID to map the proximitome of MGAT3, a glycosyltransferase responsible for the biosynthesis of the bisecting GlcNAc structure, in HEK293T cells. This approach enriched 116 and 189 proteins, respectively, identifying 17 common substrates shared with bisecting GlcNAc-bearing proteome obtained via intact glycopeptide enrichment methods. Gene ontology analysis revealed that the enriched proteins were predominantly localized in the exosome, endoplasmic reticulum, and Golgi apparatus, consistent with subcellular localization of MGAT3 substrates. Notably, four novel substrates, GOLM2, CCDC134, ASPH, and ERO1A, were confirmed to bear bisecting GlcNAc modification, validating the utility of the proximity labeling method. Furthermore, we observed that bisecting GlcNAc modification inhibits breast cancer progression by promoting the degradation of α-galactosidase A (GLA). These findings demonstrate the efficacy of proximity labeling in identifying glycosyltransferase substrates and provide insights into the functional impact of bisecting GlcNAc modification.

## 1. Introduction

Glycosylation is regulated by hundreds of glycosyltransferases and plays a key role in various biological processes, such as drug resistance, viral infection, and immune response [1,2,3,4]. Therefore, identifying glycosyltransferase substrates and clarifying their functions are essential for disease diagnosis and treatment. To facilitate site-specific glycoproteomics analysis, several methods for enriching intact glycopeptides have been developed. Techniques such as hydrophilic interaction liquid chromatography (HILIC) and lectin affinity allow for the identification of substrates by detecting diagnostic ions through mass spectrometry [5,6,7]. In addition, chemoenzymatic labeling that incorporates in vitro glycosylation reaction and chemical enrichment is also a powerful approach for selective substrate enrichment [8,9,10]. However, the identification of specific glycosyltransferase substrates remains a critical challenge due to the low abundance of glycopeptides, the structural complexity of glycans, and the technical difficulties in substrate enrichment. Developing innovative strategies to overcome these limitations is therefore urgently required.

MGAT3, also known as GnT-III, is a Golgi apparatus membrane-localized glycosyltransferase involved in the biosynthesis of N-glycan. It is responsible for the formation of the bisecting GlcNAc structure by transferring N-acetylglucosamine (GlcNAc) to the β-linked mannose within the N-linked core pentasaccharide. Currently, there is no targeted enrichment method for bisecting GlcNAc, except for using phaseolus vulgaris erythroagglutinin (PHA-E), a plant lectin that binds bisecting GlcNAc. However, this leads to the potential loss of non-galactosylated bisecting GlcNAc-bearing peptides during glycopeptide enrichment [11,12]. Furthermore, the complexity of glycosylation, including macro and micro heterogeneity (due to multiple glycosylation sites on the same protein or diverse glycans at a single site), complicates data acquisition and analysis, making it difficult to identify MGAT3 substrates [13,14].

Proximity labeling is an emerging technique that has been widely used in the past decade to identify biomacromolecule interactions [15,16,17]. This method employs engineered enzymes, peroxidases (APEX, APEX2, and HRP), and biotin ligases (BioID, BioID2, BASU, TurboID, and miniTurbo) [18]. These enzymes covalently attach biotin to nearby amino acid residues, allowing proteins of interest to be enriched by streptavidin beads and identified via mass spectrometry. Proximity labeling excels at detecting weak, transient or area-restricted protein–protein interaction in living cells, in contrast to traditional affinity purification methods [18,19]. Moreover, enzyme-catalyzed proximity labeling not only identifies spatiotemporal interactome, but also helps dissect the membrane proteome of organelle contact sites using split enzymes with higher specificity [20,21,22,23]. It has also been used to monitor protein trafficking within and between cells [24].

In this study, we used two biotin ligases, BASU and TurboID, to label the MGAT3 proximitome and enrich MGAT3 substrates in HEK293T cells. The MGAT3 proximitome shares 17 MGAT3 substrates with the bisecting GlcNAc-bearing proteome identified by Oasis MAX and zwitterionic HILIC (ZIC-HILIC) enrichment. Four candidate substrates, GOLM2, CCDC134, ASPH, and ERO1A, which were exclusively present in the MGAT3 proximitome, were confirmed to be modified by bisecting GlcNAc. This demonstrates that proximity labeling is an efficient tool for identifying MGAT3 substrates. Furthermore, we explored the impact of bisecting GlcNAc modification on GLA stability and its role in breast cancer progression.

## 2. Materials and Methods

### 2.1. Plasmid Construction

DNA fragments were amplified by PrimeSTAR^®^ Max DNA Polymerase (R045A, Takara Bio, Shiga, Japan) and inserted into the corresponding vectors. The shRNA target sequences for GLA knockdown are listed in Table 1.

### 2.2. Reverse Transcription-Quantitative Polymerase Chain Reaction (RT-qPCR)

Total RNA was extracted with TRIzol reagent (RK30129, ABclonal, Wuhan, China). ABScript III RT Master Mix for qPCR with gDNA Remover (RK20429, ABclonal, Wuhan, China) was used for first-strand cDNA synthesis. Quantitative analysis was performed using Genious 2 × SYBR Green Fast qPCR Mix (No ROX) (RK21205, ABclonal, Wuhan, China). Primer sequences for RT-qPCR are listed in Table 2.

### 2.3. Cell Lines and Cell Culture

All cell lines were purchased from the Cell Bank of the Chinese Academy of Sciences (Shanghai, China). HEK293T and MDA-MB-231 cells were cultured in DMEM supplemented with 10% fetal bovine serum (FBS), 100 UI/mL penicillin, and 100 μg/mL streptomycin.

### 2.4. Construction of Gene Knockin Cell Lines

Homology-mediated end joining (HMEJ) based on CRISPR-Cas9 was used to integrate *MGAT3-BASU* and *MGAT3-TurboID* into adeno-associated virus integration site 1 (AAVS1) locus [25]. In brief, fusion sequences of *MGAT3* and *BASU* or *TurboID* were inserted into pcDNA3.1 vector using In-Fusion cloning, and subsequently CMV enhancer-CMV promoter-fusion sequences were amplified and cloned into AAVS1-puro vector containing AAVS1 left (802 bp) and right (837 bp) homology arms to generate AAVS1-MGAT3-BASU-KI and AAVS1-MGAT3-TurboID-KI donor vectors. AAVS1-KI and HP180-gAAVS1-Cas9 vectors were co-transfected into HEK293T cells [26]. After 48 h, gene knock-in cell lines were screened with 1 μg/mL puromycin and verified by PCR and Western blot.

### 2.5. Lentivirus-Mediated Stable Knockdown and Overexpression of GLA

To obtain stable knockdown and overexpression cell lines of GLA, the shRNA oligos of *GLA* were cloned into pLKO.1-puro vector and the coding sequence of *GLA* with a FLAG tag was cloned into pLVX-IRES-hygro vector. The lentivirus packaging vector psPAX2 and envelope vector pMD2.G were co-transfected into HEK293T cells along with pLVX or pLKO.1 vector (psPAX2:pMD2.G:pLVX/pLKO.1 = 3:1:4, *w*/*w*), and the supernatant containing lentivirus was harvested and filtered through 0.22 µm filter after 48 h to infect MDA-MB-231 cells. Stable knockdown cell lines were screened with 1 μg/mL puromycin, and the stable overexpression cell line was screened with 200 μg/mL hygromycin B.

### 2.6. Western Blot and Lectin Blot

Cells were washed three times with PBS and lysed with RIPA buffer (HY-K1001, MCE, Monmouth Junction, NJ, USA) containing proteinase inhibitor cocktail (C0001, TargetMol, Boston, MA, USA). Protein concentration was measured by BCA Protein Assay Kits (BCA02, Dingguo Changsheng, Beijing, China). Proteins were separated by sodium dodecyl sulfate-polyacrylamide gel electrophoresis (SDS-PAGE) and subsequently transferred to polyvinylidene fluoride (PVDF) membrane (BSP0161, Pall, Port Washington, NY, USA). After blocking with 3% BSA or 5% skim milk powder in TBST, the membranes were incubated with the specific antibodies, biotinylated PHA-E or HRP-conjugated streptavidin. The membranes were then immersed in ECL substrate solution and imaged using a chemiluminescent imaging system (Tanon 5200 Multi, Tanon, Shanghai, China).

### 2.7. CCK-8 Assay

Cells were seeded into a 96-well plate, and, at the indicated time, the culture medium was replaced with fresh complete medium containing 10% CCK-8 solution (E1CK-000208, EnoGene, Nanjing, China). After incubating for 1 h at 37 °C with 5% CO_2_, absorbance at 450 nm was measured using microplate reader (HBS-1096A, DeTie, Nanjing, China).

### 2.8. Transwell Assay

Cells were seeded into the upper chamber of a Transwell insert with an 8 μm pore membrane insert (TCS020024, JETBIOFIL, Guangzhou, China). A total of 500 µL complete medium was added to the lower chamber. After 48 h, cells that migrated to the bottom of the membrane were fixed with 4% paraformaldehyde for 15 min, stained with 0.1% crystal violet for 15 min, and imaged using a microscope.

### 2.9. Flow Cytometry

For proliferation analysis, the iClick™ EdU Andy Fluor 647 Flow Cytometry Assay Kit (A008, ABP Biosciences, Rockville, MD, USA) was used. Briefly, cells were incubated with 10 µM EdU for 12 h, fixed with 4% paraformaldehyde for 15 min, and permeated with 0.2% Triton X-100 for 10 min. Cells were then incubated with Click-iT reaction cocktail for 30 min in the dark and detected by flow cytometer (NovoCyte 2060R, ACEA Biosciences, San Diego, CA, USA).

For cell apoptosis analysis, cells were harvested, washed three times with PBS, and resuspended with 100 µL binding buffer (422201, BioLegend, San Diego, CA, USA). A total of 5 µL APC Annexin V (640920, BioLegend, San Diego, CA, USA) and 5 µL 7-AAD (420404, BioLegend, San Diego, CA, USA) were added to each sample and incubated for 15 min in the dark. Cells were detected by flow cytometer after adding 400 µL binding buffer.

### 2.10. On-Beads Digestion

Proteins were extracted as described above. A total of 5 mg of total protein per biological replicate was used for the enrichment of biotinylated proteins, and three biological replicates were performed. Streptavidin magnetic beads (HY-K0208-1 mL, MCE, Monmouth Junction, NJ, USA) were washed three times with 50 mM ammonium bicarbonate (ABC) and incubated with cell lysate for 1 h at room temperature (RT). The magnetic beads were washed twice with RIPA lysis buffer and then three times with 50 mM ABC. After magnetic separation, the supernatant was removed, and the proteins were reduced with 200 μL 50 mM ABC containing 10 mM dithiothreitol (DTT) for 1 h at 37 °C with shaking. Proteins were alkylated with 20 mM iodoacetamide (IAA) for 30 min in the dark at RT with shaking and then 10 mM DTT was added again. After 30 min, samples were digested with 1 μg trypsin for 13–16 h at 37 °C with rotation. The next day, the digest supernatant was transferred to new tubes and the beads were washed twice with 100 μL 50 mM ABC. The mixture combining the digest supernatant and the washes was acidified with formic acid (FA) to pH < 3, centrifuged at 14,000× *g* for 10 min to remove insoluble pellet, desalted with C18 StageTips, and evaporated using a rotational vacuum concentrator (RVC 2-18 CDplus, Martin Christ, Osterode am Harz, Germany).

### 2.11. Intact Glycopeptide Enrichment

Cells cultured in the 10 cm dish were washed three times with PBS and lysed with 8M urea in 50 mM ABC. After incubation for 20 min and ultrasonic treatment (10 s on and 10 s off, six cycles in total), cell lysate was centrifuged at 14,000× *g* for 10 min and the pellet was removed. A total of 1 mg of protein sample was reduced and alkylated as described in on-beads digestion. Samples were diluted eight times with 50 mM ABC. Trypsin was added to each sample (1:50, *w*/*w*) and incubated for 13–16 h at 37 °C with rotation. Samples were acidified with trifluoroacetic acid (TFA) to pH < 3, centrifuged at 14,000× *g* for 10 min to remove insoluble pellet, desalted using hydrophile–lipophile balance (HLB) extraction cartridge (WAT094225, Waters, Milford, MA, USA), and evaporated. Subsequently, intact glycopeptides were enriched by Oasis MAX extraction cartridge (186001883, Waters, Milford, MA, USA) and ZIC-HILIC StageTips.

### 2.12. Immunoprecipitation-Mass Spectrometry

Protein extraction was performed as described above. A total of 1 μg of primary antibody was added to 2 mg protein and incubated for 1 h at 4 °C with rotation. In total, 20 μL Protein A + G agarose was added to samples and incubated overnight at 4 °C with rotation. Samples were centrifuged at 1000× *g* for 5 min at 4 °C and supernatant was removed. Agarose was washed four times with PBS and supernatant was carefully removed after finishing the last wash. Samples were mixed with 40 μL 1 × protein loading buffer and then denatured by dry bath for 5 min at 98 °C. Proteins were separated by SDS-PAGE and polyacrylamide gels were incubated with coomassie brilliant blue R-250 staining solution for 1 h at RT. After gels were destained until background was nearly clear, gel pieces of interest were excised, transferred to new tubes, and destained with 30% acetonitrile (ACN) plus 50 mM ABC. Proteins were reduced and alkylated during repeated dehydration and rehydration. Following removal with 100% ACN, 1 μg trypsin in 50 mM ABC was added to samples, and they were incubated for 13–16 h at 37 °C. Digested peptides were extracted twice with 150 μL 60% ACN/0.1% TFA by bath sonication, evaporated, purified with C18 StageTips, and then evaporated again.

### 2.13. Liquid Chromatography Coupled to Tandem Mass Spectrometry

Mass spectrometry data were acquired in a data-dependent mode using an EASY-nLC 1200 system coupled to a Q Exactive Plus mass spectrometer (Thermo Fisher Scientific, Waltham, MA, USA). Desalted peptides were redissolved in 0.1% FA and separated on a liquid chromatography column (C18-AQ, 1.9 µm, PF360-75-10-N-5, 20 cm column length). The mobile phase consisted of mobile phase A (0.1% FA in H_2_O) and mobile phase B (80% ACN/0.1% FA). For a 120 min gradient separation, mobile phase B at a flow rate of 200 nL/min was gradually increased from 3% to 32% for 95 min, 32% to 100% for 10 min, and maintained at 100% for 15 min. The full scan range was 355–1700 *m*/*z* at a resolution of 70,000. The automatic gain control (AGC) target and maximum injection time were set to 5 × 10^5^ and 100 ms, respectively. MS/MS scans were acquired in an Orbitrap with a 17,500 resolution. HCD-based fragmentation was performed with 27% normalized collisional energy for peptides and 20–30–40% stepped collisional energy for glycopeptides.

### 2.14. Data Analysis

Bioinformatic analysis was performed using the OmicStudio tools, a free online platform (https://www.omicstudio.cn/tool, accessed on 3 August 2020). Raw files for mass spectrometry were processed using Proteome Discoverer (PD) v2.2.0.388, Perseus v2.0.7.0, and Glyco-Decipher v1.0.4. Mass spectra were extracted from Xcalibur v4.1.31.9. The gray scale intensity of Western blot was measured by ImageJ. GraphPad Prism 7 was used for statistical analysis.

## 3. Results

### 3.1. Validation of Proximity Labeling Tools in Living Cells

**Figure 1:**
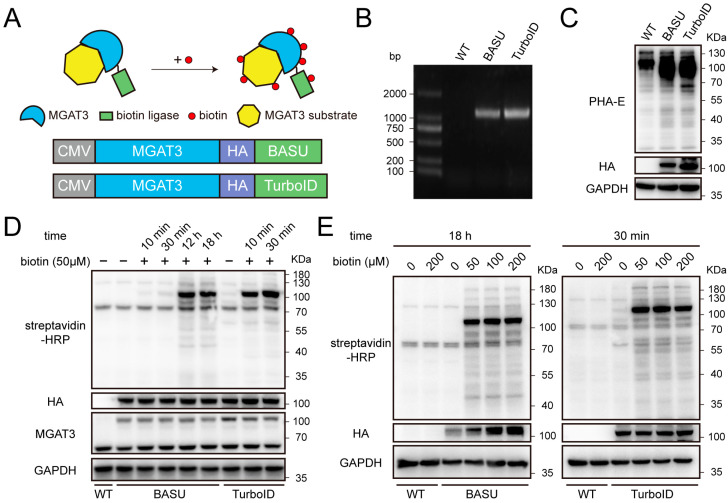
Construction and validation of proximity labeling tools in HEK293T cells. (**A**) Schematic illustration of the biotinylation labeling for MGAT3 substrates (top) and the construction of two fusion proteins, with an HA tag inserted between MGAT3 and biotin ligase (bottom). (**B**) Agarose gel electrophoresis confirming knock-in fragments at AAVS1 locus. Abbreviations: WT, wild-type; BASU, MGAT3-BASU knock-in; TurboID, MGAT3-TurboID knock-in. (**C**) Lectin blot analysis of the level of bisecting GlcNAc modification. (**D**) Validation of the level of protein biotinylation under time-dependent biotin incubation. (**E**) Validation of the level of protein biotinylation under concentration-dependent biotin incubation.

The biotin reactive species catalyzed by the fusion proteins can covalently label MGAT3 substrates, as well as proteins that interact with or are located nearby (Figure 1A). In order to map the MGAT3 proximitome in living cells, two biotin ligases with different enzymatic properties, BASU and TurboID, were fused to full-length MGAT3 protein (Figure 1A and Table S1). These fusion constructs were successfully integrated into the AAVS1 locus of HEK293T cells using CRISPR-Cas9 technology (Figure 1B). The expression of exogenous MGAT3 significantly elevated the level of bisecting GlcNAc modification, indicating that the function of MGAT3 remained intact despite being fused with the biotin ligase (Figure 1C). While previous studies have established recommended biotin concentrations and incubation times for different biotin ligases, we revisited these parameters due to potential changes in protein expression and localization in this study [18,27,28]. For MGAT3-BASU cells, 18 h of incubation with 50 μM biotin was sufficient to label proteins proximal to MGAT3, whereas MGAT3-TurboID cells only required 30 min (Figure 1D). In addition, a concentration-dependent analysis indicated that biotin concentrations between 50 μM and 200 μM did not significantly affect labeling intensity (Figure 1E). Based on these findings, 50 μM biotin was chosen as the optimal concentration, with the incubation times set at 18 h for MGAT3-BASU cells and 30 min for MGAT3-TurboID cells for the purpose of mapping the MGAT3 proximitome.

### 3.2. Profiling of the MGAT3 Proximitome Labeled by BASU and TurboID

**Figure 2:**
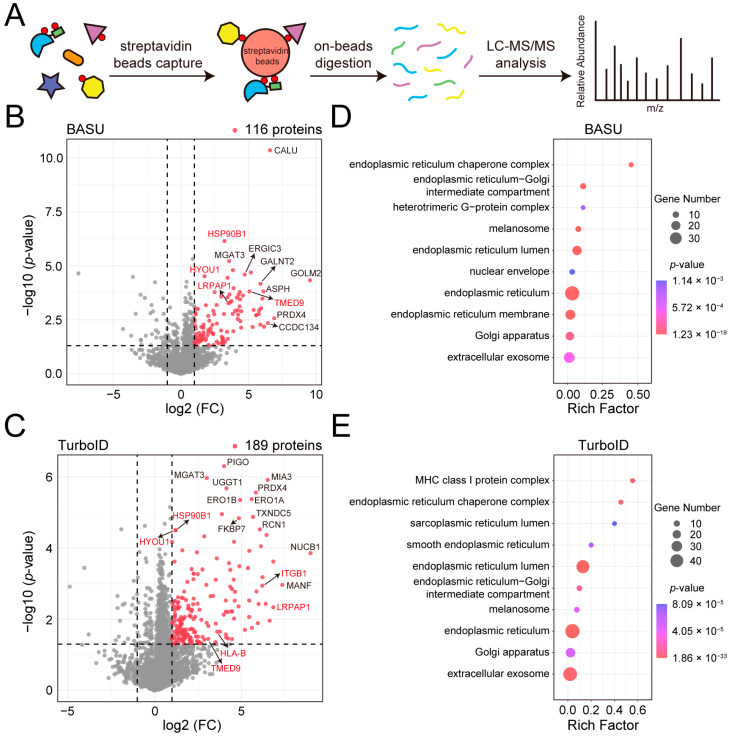
Analysis of the MGAT3 proximitome labeled by BASU and TurboID. (**A**) Flowchart for enrichment and identification of the MGAT3 proximitome. (**B**) Volcano plot highlighting 116 significantly enriched proteins in biotin-treated MGAT3-BASU cells (*p*-value < 0.05 and fold change > 2). Known MGAT3 substrates are highlighted in red. (**C**) Volcano plot highlighting 189 significantly enriched proteins in biotin-treated MGAT3-TurboID cells (*p*-value < 0.05 and fold change > 2). Known MGAT3 substrates are highlighted in red. (**D**) Gene ontology (GO) analysis of the MGAT3-BASU proximitome. (**E**) GO analysis of the MGAT3-TurboID proximitome.

To identify the MGAT3 proximitome labeled by BASU and TurboID, biotinylated proteins were enriched, digested on streptavidin beads, and analyzed by a label-free quantification (LFQ) approach (Figure 2A). In MGAT3-BASU cells treated with biotin, 116 proteins were significantly enriched compared to the control group without biotin treatment (Figure 2B and Table S2). In MGAT3-TurboID cells treated with biotin, 189 proteins were significantly enriched (Figure 2C and Table S3). In addition to the decoy protein MGAT3, several known MGAT3 substrates were detected in the MGAT3 proximitome, including LRPAP1, TMED9, and ITGB1 (Figure 2B,C and Figure S1) [29,30]. Gene ontology (GO) analysis revealed that these enriched proteins were predominantly localized in the exosome, endoplasmic reticulum, and Golgi apparatus (Figure 2D,E). N-glycosylation is initiated in the endoplasmic reticulum and further processed in the Golgi apparatus. The mature N-glycosyltransferase substrates can then be transported back to the endoplasmic reticulum, or reside in the Golgi apparatus, or transported to other locations, such as the plasma membrane, lysosome, and exosome. Therefore, the localization of these enriched proteins was in line with that of MGAT3 substrates. Nevertheless, some proteins in the MGAT3 proximitome may be labeled during fusion proteins’ processing and transport from the endoplasmic reticulum to the Golgi apparatus.

### 3.3. Identification of Novel MGAT3 Substrates in the MGAT3 Proximitome

**Figure 3:**
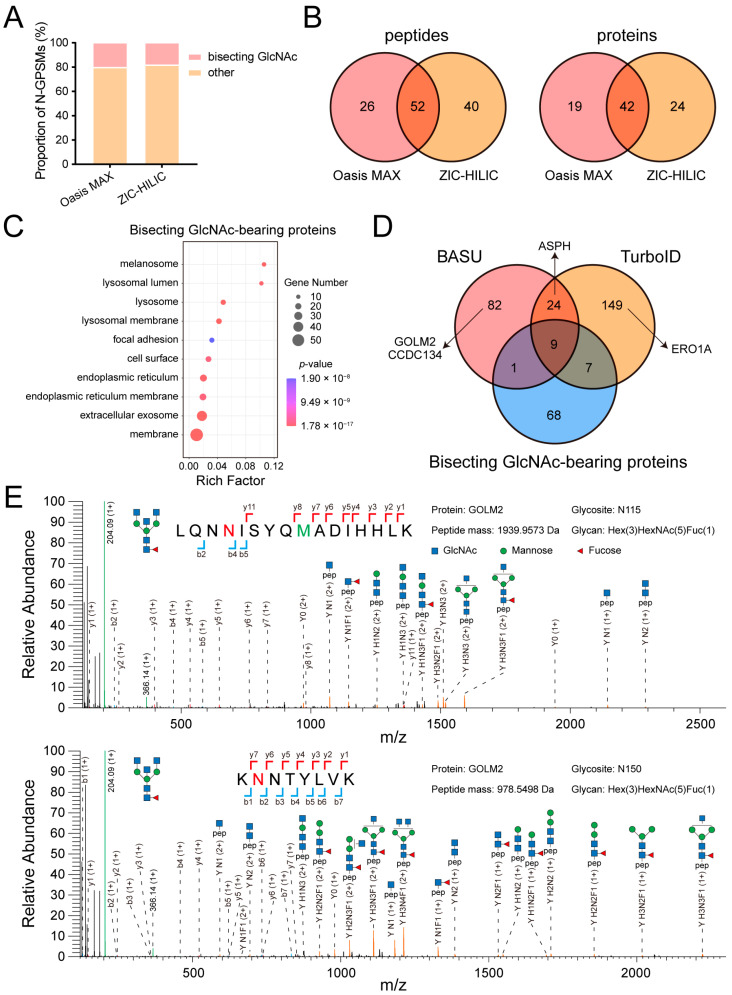
Identification of bisecting GlcNAc-bearing proteins and validation of novel MGAT3 substrates. (**A**) Stacked bar chart showing the proportion of bisecting GlcNAc-modified N-GPSMs relative to total N-GPSMs. (**B**) Venn diagram comparing bisecting GlcNAc-bearing peptides and proteins enriched by Oasis MAX and ZIC-HILIC. (**C**) GO analysis of 85 identified proteins bearing bisecting GlcNAc modification. (**D**) Venn diagram comparing two MGAT3 proximitomes with the identified bisecting GlcNAc-bearing proteins. (**E**) Representative MS/MS spectrum of GOLM2 glycopeptides bearing bisecting GlcNAc. The N-glycosites are highlighted in red and oxidation-methionine is marked in green.

To enrich intact glycopeptides from HEK293T cells, two solid phase extraction materials, Oasis MAX and ZIC-HILIC, were employed, followed by the large-scale N-glycoproteome identification via Glyco-Decipher [31]. There was minimal difference in the proportion of N-glycopeptide-spectrum matches (N-GPSMs) containing bisecting GlcNAc, as well as in the number of bisecting GlcNAc-bearing proteins identified by the two enrichment methods (Figure 3A,B). Oasis MAX identified 78 unique bisecting GlcNAc-bearing peptides corresponding to 61 proteins, while ZIC-HILIC identified 92 unique bisecting GlcNAc-bearing peptides corresponding to 66 proteins, with 42 proteins overlapping between the two methods (Figure 3B). Similarly to the MGAT3 proximitome, all 85 bisecting GlcNAc-bearing proteins were significantly localized in the membrane, exosome, and endoplasmic reticulum (Figure 3C).

We further compared bisecting GlcNAc-bearing proteome with the two MGAT3 proximitomes and found that 17 MGAT3 substrates were labeled by either BASU and TurboID (Figure 3D and Table S4). To identify novel MGAT3 substrates, we selected four candidates, GOLM2, CCDC134, ASPH, and ERO1A, from the top 30 proteins present only in the MGAT3 proximitome, and verified their bisecting GlcNAc modification by using immunoprecipitation-mass spectrometry (IP-MS) (Figure 3D and Figure S1). The canonical sequence of GOLM2, as listed in the UniProt database, includes three potential N-glycosites at N115, N150, and N364. However, in this study, an isoform of GOLM2 (UniProt ID: Q6P4E1-2) was identified from HEK293T cells, which only left two glycosites remaining, N115 and N150. The presence of diagnostic ions [Y+H1N3] or [Y+H1N3F1] confirmed that both of them contain bisecting GlcNAc structure (Figure 3E). In addition, three other proteins were also confirmed to be modified by bisecting GlcNAc (Figure S2).

### 3.4. Bisecting GlcNAc-Facilitated GLA Degradation in Breast Cancer Cells

**Figure 4:**
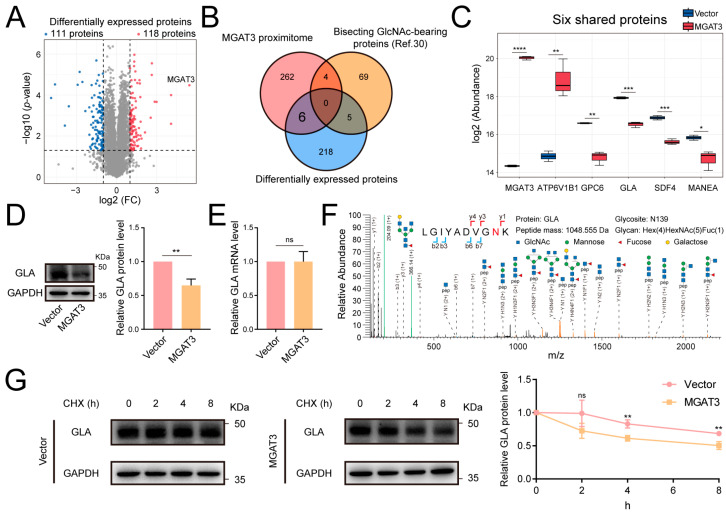
Effect of the bisecting GlcNAc modification on GLA stability. (**A**) Volcano plot showing differently expressed proteins between MGAT3/231 cells and Vector/231 cells. (**B**) Venn diagram comparing MGAT3 proximitome, differently expressed proteins, and bisecting GlcNAc-bearing proteins identified in MDA-MB-231 cells by PHA-E affinity enrichment [30]. (**C**) Protein abundance of six proteins shared by the MGAT3 proximitome and differently expressed proteins. (**D**) Western blot analysis of GLA protein level. (**E**) Quantitative analysis of GLA mRNA level. (**F**) Representative MS/MS spectrum of GLA glycopeptide bearing bisecting GlcNAc. The N-glycosite is highlighted in red. (**G**) Western blot analysis of GLA stability under time-dependent CHX treatment (100 μM). ns, not significant; *, *p* < 0.05; **, *p* < 0.01; ***, *p* < 0.001; ****, *p* < 0.0001.

Our previous research has demonstrated that increased bisecting GlcNAc modification inhibits the development of breast cancer, and the influence of glycosylation on protein stability has been widely reported [30,32,33,34,35]. To investigate potential MGAT3 substrates with significantly altered protein levels in MDA-MB-231 cells and their role in breast cancer, a proteomic analysis was performed using MDA-MB-231 cells that were engineered to overexpress MGAT3 (MGAT3/231) [32]. In this analysis, 118 proteins were significantly up-regulated, and 111 proteins were significantly down-regulated in MGAT3/231 cells compared to Vector/231 cells (Figure 4A). When comparing the proteomics data with the MGAT3 proximitome, six proteins were found to be shared between the MGAT3 proximitome and the differentially expressed proteins, which were not identified by PHA-E enrichment in MDA-MB-231 cells (Figure 4B,C) [30].

One notable finding was the significant reduction in the abundance of α-galactosidase A (GLA) protein in MGAT3/231 cells, suggesting that GLA’s stability might be regulated by bisecting GlcNAc modification (Figure 4C). Upon overexpression of MGAT3, we observed a decrease in GLA protein level but no change in mRNA level, and mass spectrometry analysis further confirmed that GLA was modified by bisecting GlcNAc (Figure 4D,F). Moreover, GLA exhibited greater stability in Vector/231 cells than in MGAT3/231 cells when treated with cycloheximide (CHX), implying that the enhanced bisecting GlcNAc modification promotes GLA degradation (Figure 4G).

### 3.5. Bisecting GlcNAc Inhibited the Tumor-Promoting Effect of GLA in Breast Cancer Cells

**Figure 5:**
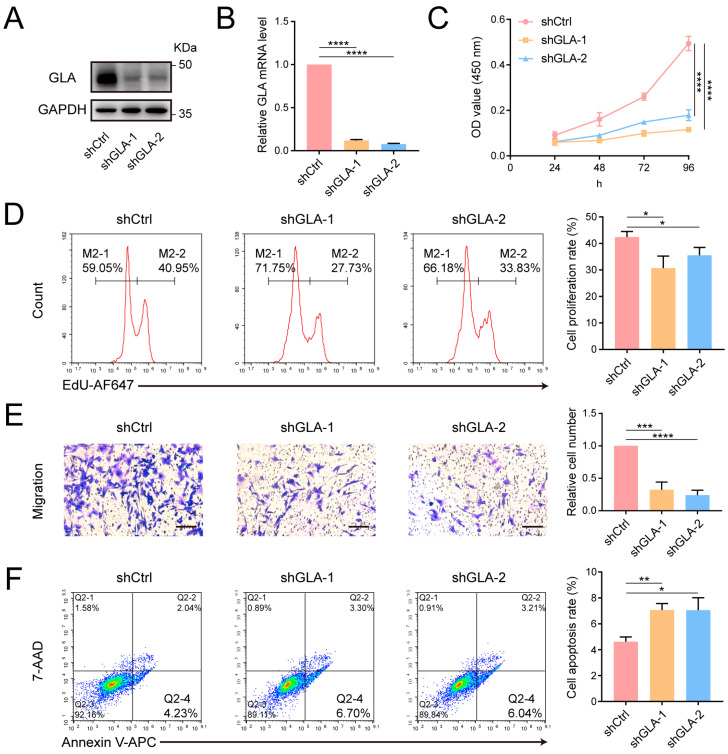
Effect of GLA knockdown on the phenotype of MDA-MB-231 cells. (**A**) Western blot analysis confirming reduced GLA protein level after shRNA transfection. (**B**) Quantitative analysis of GLA mRNA level. (**C**) CCK-8 assay evaluating cell proliferation. (**D**) Flow cytometry analysis evaluating cell proliferation. (**E**) Transwell assay measuring cell migration. Scale bar: 200 μm. (**F**) Flow cytometry analysis evaluating cell apoptosis. *, *p* < 0.05; **, *p* < 0.01; ***, *p* < 0.001; ****, *p* < 0.0001.

**Figure 6:**
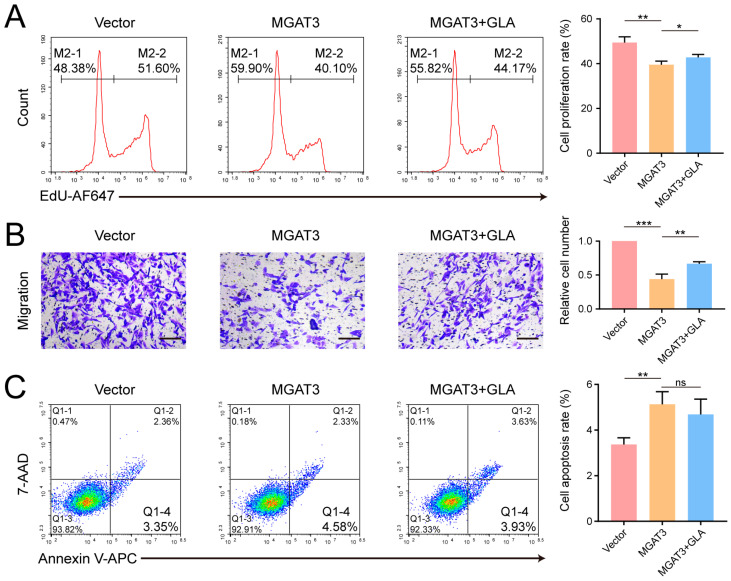
Effect of GLA overexpression on the phenotype of MGAT3/231 cells. (**A**) Flow cytometry analysis evaluating cell proliferation. (**B**) Transwell assay evaluating cell migration. Scale bar: 200 μm. (**C**) Flow cytometry analysis evaluating cell apoptosis. ns, not significant; *, *p* < 0.05; **, *p* < 0.01; ***, *p* < 0.001.

GLA is a lysosomal enzyme responsible for hydrolyzing terminal galactose residues. Deficient GLA activity generally leads to Fabry disease, but its role in tumor development has been scarcely studied [36]. To investigate the role of GLA in breast cancer cells, we constructed MDA-MB-231 cell lines with stable GLA knockdown using shRNA lentivirus (Figure 5A,B) and found that GLA knockdown significantly suppressed cell proliferation in both CCK-8 and EdU assay, reduced cell migration, and promoted apoptosis (Figure 5C–F). Furthermore, we stably expressed GLA-FLAG in MGAT3/231 cells and observed that exogenous GLA enhanced cell proliferation and migration but had little effect on apoptosis (Figure 6A–C and Figure S3). Taken together, these results suggest that bisecting GlcNAc modification inhibits breast cancer progression by promoting GLA degradation.

## 4. Discussion

The proximity labeling strategy is valuable for deciphering interactions between biomolecules. Due to the typically weak interaction between glycan-binding proteins and their ligands, peroxidase-based proximity labeling has been successfully applied to identify ligands of galectin-3, siglec-2, and siglec-15 [37,38]. Another study combined the BioID with the tetratricopeptide repeat (TPR) domain of OGT, identifying 115 OGT-TPR interacting proteins, 46 of which were previously recognized as OGT substrates in omics studies [39]. Furthermore, a GlycoID tool, based on the TurboID proximity labeling system, enables targeted biotin labeling of O-GlcNAc-modified proteins within cells [40]. Although these OGT studies did not further validate the presence of O-GlcNAc modifications on potential substrates, accumulating evidence supports that proximity labeling can effectively identify both kinase and ubiquitin ligase substrates [41,42,43].

In this study, we investigated the efficacy of proximity labeling for identifying substrates of glycosyltransferases using MGAT3 as a model. Because the cytotoxicity of H_2_O_2_ constrains the long-term application of peroxidases in live cells, we used two biotin ligases, BASU and TurboID, to label proteins in proximity to MGAT3. Notably, the high activity of TurboID can result in low-level biotinylation of proteins even without added biotin, consistent with prior observations of TurboID’s broader labeling range [44]. Therefore, we implemented careful temporal control over TurboID labeling to maximize the signal-to-noise ratio (Figure 1D,E). Research has shown a positive correlation between protein biotinylation level and biotin concentration, yet, in our experiments, increasing the biotin concentration from 50 μM to 200 μM did not produce significant changes in the total biotinylation level in cells expressing MGAT3-BASU or MGAT3-TurboID [45,46]. This finding indicates that 50 μM biotin is sufficient for experiments involving CRISPR-Cas9-mediated expression of biotin ligases (Figure 1E).

The fusion of BASU and TurboID with MGAT3 led to the identification of 116 and 189 enriched proteins, respectively (Figure 2B,C). GO analysis revealed that both MGAT3 proximitomes were predominantly localized in the exosome, endoplasmic reticulum, and Golgi apparatus, consistent with the subcellular localization of MGAT3 substrates identified by intact glycopeptide enrichment (Figure 2D,E and Figure 3C). Comparing the two MGAT3 proximitomes with bisecting GlcNAc-modified proteins, we identified 17 common MGAT3 substrates labeled by BASU and TurboID, including HYOU1, TMED9, and ITGB1 (Figure 3D and Table S4). In addition, we also noticed that MGAT3-BASU and MGAT3-TurboID proximitomes overlap only a small fraction of their proteins, which may be due to differences in structure and enzyme activity between BASU and TurboID (Figure 3D). This variability aligns with findings from a prior study comparing split-TurboID and Contact-ID, where differences in labeling time and enzyme activity also influenced the results of proteomic analyses at the ER-mitochondria contact sites [23]. To confirm that proximity labeling can identify novel MGAT3 substrates, we validated four exclusive candidate substrates (GOLM2, CCDC134, ASPH, and ERO1A) using IP-MS, and all were found to bear bisecting GlcNAc (Figure 3E and Figure S2).

Similarly to phosphorylation and ubiquitination, glycosylation can involve multiple glycosyltransferases, such as mucin-type O-glycosylation initiation, and the substrates of these glycosyltransferases remain poorly defined [47]. Proximity labeling holds promise for identifying specific isozyme substrates. However, this method also has limitations. Taking our study as an example, the MGAT3 proximitome may contain non-substrate proteins in close physical proximity to MGAT3. To improve specificity, enzymatically inactive MGAT3 mutants could be used as negative controls. Reducing background interference, such as organelle structural proteins, will be critical for accurate identification of MGAT3 substrates [48]. Overall, this method provides a spatially guided pool of potential MGAT3 substrates. Compared to traditional glycan-based enrichment methods, proximity labeling enables the identification of MGAT3 substrates that may be unrecognizable by PHA-E or undetectable by mass spectrometry due to the low abundance of bisecting GlcNAc-bearing peptides.

Aberrant expression of bisecting GlcNAc has been observed in various diseases, including cancer, Alzheimer’s disease, and diabetes [49,50,51]. Our previous studies demonstrated that bisecting GlcNAc plays a tumor-suppressive role in breast cancer, as evidenced by the inhibition of tumor growth, metastasis, and drug resistance [30,32,52]. To further investigate the role of bisecting GlcNAc in breast cancer, we compared the MGAT proximitome with differentially expressed proteins following MGAT3 overexpression, identifying six overlapping proteins in both datasets, which were undetectable by PHA-E enrichment. We further confirmed that GLA was modified by bisecting GlcNAc and found that MGAT3 overexpression accelerated GLA degradation. One possible explanation is that the addition of bisecting GlcNAc directly promotes the interaction between GLA and degradation pathway-associated proteins. However, the bisecting GlcNAc conformation generally hinders the formation of branched GlcNAc, including β1,6-GlcNAc branch produced by MGAT5 and the terminal modification of N-glycans, because other N-glycosyltransferases favor that glycans without bisecting GlcNAc as substrates [53,54,55]. Therefore, the stability of GLA may also be supported by branched GlcNAc or terminal modification, which is compromised by bisecting GlcNAc.

Notably, GLA is highly expressed in several cancers, including breast cancer [56]. Additionally, Fabry patients, caused by mutations in the GLA gene, exhibit a slightly reduced cancer rate [57]. In this study, we found that GLA knockdown suppressed proliferation and migration while promoting apoptosis in MDA-MB-231 cells, indicating that bisecting GlcNAc modification inhibits breast cancer progression by promoting GLA degradation. However, further experiments, such as investigating the specific degradation pathways of GLA, are needed to rigorously validate this conclusion and provide deeper mechanistic insights.

## 5. Conclusions

Despite its limitations, the proximity labeling strategy is effective for identifying potential MGAT3 substrates, providing a basis for investigating substrates of other glycosyltransferases. Additionally, our findings suggest that bisecting GlcNAc inhibits breast cancer progression by enhancing GLA degradation.

## Figures and Tables

**Table 1:** The shRNA target sequences for GLA knockdown.

Name	Sequence (5′–3′)
shGLA-1	CTGCAATCACTGGCGAAATTT
shGLA-2	TGCTCCTTTATTCATGTCTAA

**Table 2:** Primer sequences for RT-qPCR.

Name	Sequence (5′–3′)
GAPDH-Forward	GGAGCGAGATCCCTCCAAAAT
GAPDH-Reverse	GGCTGTTGTCATACTTCTCATGG
GLA-Forward	TTGGATACTACGACATTGATGCC
GLA-Reverse	TTCTGCCAGTCCTATTCAGGG

## Data Availability

The data that support the findings of this study are available from the corresponding authors upon reasonable request.

## Supplementary Materials

The following supporting information can be downloaded at: https://www.mdpi.com/article/10.3390/cells14020103/s1, Figure S1: Abundance of the top 30 proteins in the MGAT3 proximitome; Figure S2: Representative MS/MS spectra of novel MGAT3 substrates bearing bisecting GlcNAc; Figure S3: Western blot analysis of exogenous GLA-FLAG expression; Table S1: The complete protein sequences of MGAT3-BASU and MGAT3-TurboID; Table S2: A comprehensive list of 116 significantly enriched proteins in MGAT3-BASU cells treated with biotin; Table S3: A comprehensive list of 189 significantly enriched proteins in MGAT3-TurboID cells treated with biotin. Table S4: 17 MGAT3 substrates labeled by BASU and TurboID. The complete dataset for proximity labeling and glycopeptide enrichment was included in the Supplementary MS Tables.
