# Supplementary material for: Proximity Labeling-Based Identification of MGAT3 Substrates and Revelation of the Tumor-Suppressive Role of Bisecting GlcNAc in Breast Cancer via GLA Degradation
Source: Cells. 2025 Jan 12;14(2):103. doi: 10.3390/cells14020103 (PMC11764451; doi:10.3390/cells14020103)
Supplement: Supplementary file 1 [file cells-14-00103-s001.zip › Supplementary Figures and Tables.pdf]

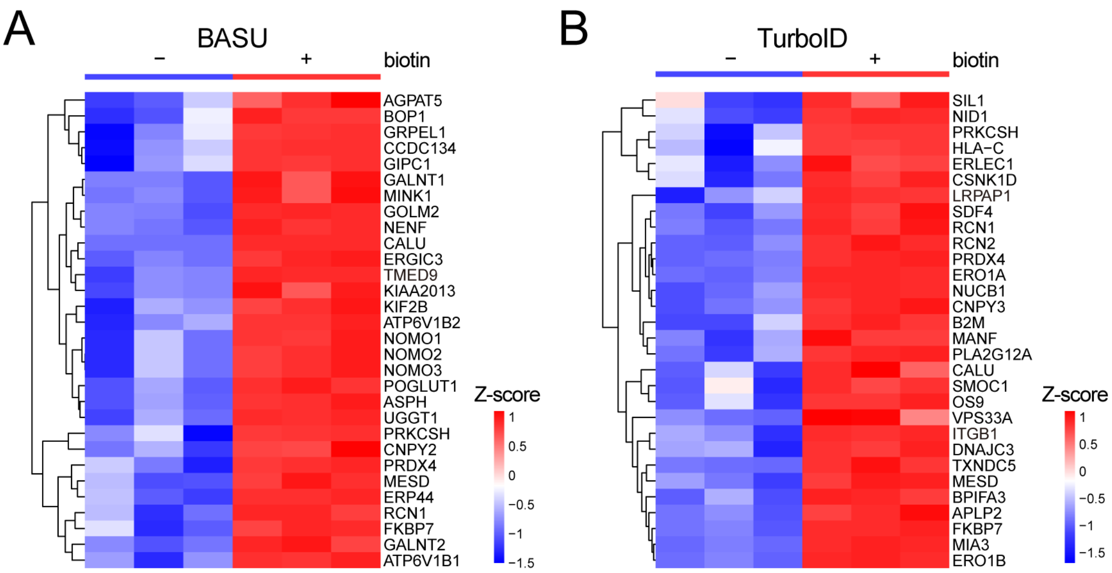

Figure S1. Abundance of the top 30 proteins in the MGAT3 proximitome. (**A**) Heatmap of the top 30 proteins (ranked by fold change) in the MGAT3-BASU proximitome. (**B**) Heatmap of the top 30 proteins (ranked by fold change) in the MGAT3-TurboID proximitome.

Table S4. 17 MGAT3 substrates labeled by BASU and TurboID.

Uniprot accession	Protein	Bisecting GlcNAc-bearing glycosite(s)
O43852	CALU	N131
P14625	HSP90B1	N217
Q09327	MGAT3	N141, N241, N259
Q9Y4L1	HYOU1	N862, N869
Q9BVK6	TMED9	N125
Q8TEM1	NUP210	N337, N681
P06280	GLA	N139, N192
Q6ZXV5	TMTC3	N541
Q15293	RCN1	N53
P10586	PTPRF	N721
O96005	CLPTM1	N295
P05556	ITGB1	N212
Q9UNW1	MINPP1	N242
P15586	GNS	N362
Q02809	PLOD1	N197
Q8N766	EMC1	N913
P04062	GBA1	N309

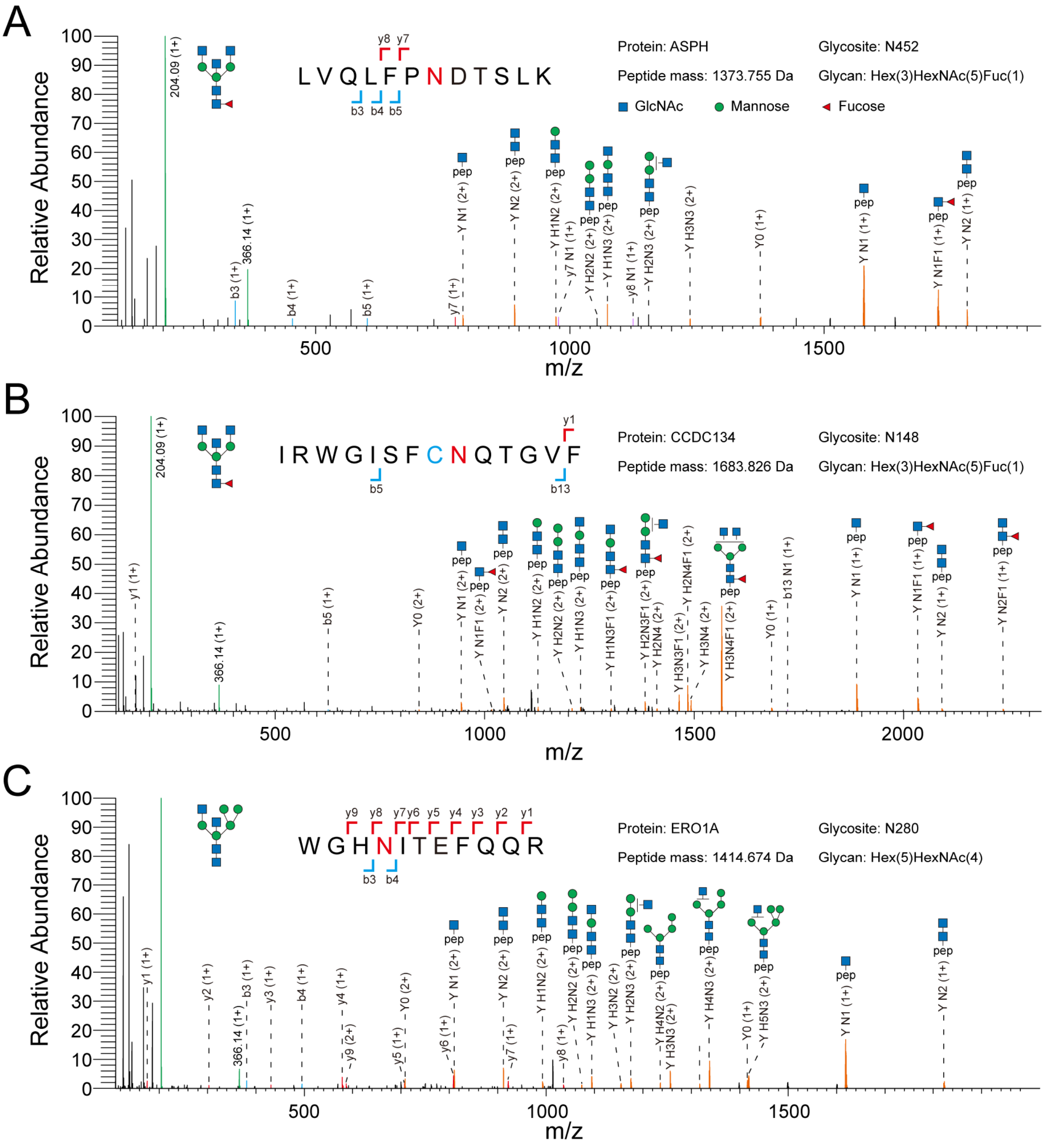

Figure S2. Representative MS/MS spectra of novel MGAT3 substrates bearing bisecting GlcNAc. (**A**) Representative MS/MS spectrum of ASPH glycopeptide bearing bisecting GlcNAc. (**B**) Representative MS/MS spectrum of CCDC134 glycopeptide bearing bisecting GlcNAc. (digested with chymotrypsin). (**C**) Representative MS/MS spectrum of ERO1A glycopeptide bearing bisecting GlcNAc. The N-glycosites are highlighted in red and carbamidomethyl-cysteine is marked in blue.

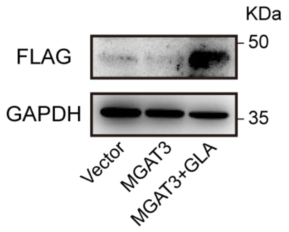

Figure S3. Western blot analysis of exogenous GLA-FLAG expression.
